# Characteristics, risk factors, and outcomes related to Zika virus infection during pregnancy in Northeastern Thailand: A prospective pregnancy cohort study, 2018–2020

**DOI:** 10.1371/journal.pntd.0012176

**Published:** 2024-05-17

**Authors:** Jurai Wongsawat, Somsak Thamthitiwat, Victoria J. Hicks, Sumonmal Uttayamakul, Phanthaneeya Teepruksa, Pongpun Sawatwong, Beth Skaggs, Philip A. Mock, John R. MacArthur, Inthira Suya, Patranuch Sapchookul, Paul Kitsutani, Terrence Q. Lo, Apichart Vachiraphan, Ekachai Kovavisarach, Chulwoo Rhee, Pamorn Darun, Kamol Saepueng, Chamnan Waisaen, Doungporn Jampan, Pravit Sriboonrat, Buncha Palanuwong, Punchawee Sukbut, Darin Areechokchai, Chakrarat Pittayawonganon, Sopon Iamsirithaworn, Emily Bloss, Carol Y. Rao

**Affiliations:** 1 Thailand Ministry of Public Health, Department of Disease Control, Nonthaburi, Thailand; 2 Thailand Ministry of Public Health–US Centers for Disease Control and Prevention Collaboration, Nonthaburi, Thailand; 3 US Centers for Disease Control and Prevention, Division of Global Health Protection, Atlanta, Georgia, United States of America; 4 Thailand Ministry of Public Health, Department of Medical Services, Nonthaburi, Thailand; 5 Bueng Kan Provincial Public Health Office, Bueng Kan, Thailand; 6 Bueng Kan Hospital, Bueng Kan, Thailand; 7 Mukdahan Provincial Public Health Office, Mukdahan, Thailand; 8 Mukdahan Hospital, Mukdahan, Thailand; Australian Red Cross Lifelood, AUSTRALIA

## Abstract

**Background:**

In response to the 2015–2016 Zika virus (ZIKV) outbreak and the causal relationship established between maternal ZIKV infection and adverse infant outcomes, we conducted a cohort study to estimate the incidence of ZIKV infection in pregnancy and assess its impacts in women and infants.

**Methodology/Principal findings:**

From May 2018-January 2020, we prospectively followed pregnant women recruited from 134 participating hospitals in two non-adjacent provinces in northeastern Thailand. We collected demographic, clinical, and epidemiologic data and blood and urine at routine antenatal care visits until delivery. ZIKV infections were confirmed by real-time reverse transcriptase polymerase chain reaction (rRT-PCR). Specimens with confirmed ZIKV underwent whole genome sequencing.

Among 3,312 women enrolled, 12 (0.36%) had ZIKV infections, of which two (17%) were detected at enrollment. Ten (83%, 3 in 2^nd^ and 7 in 3^rd^ trimester) ZIKV infections were detected during study follow-up, resulting in an infection rate of 0.15 per 1,000 person-weeks (95% CI: 0.07–0.28). The majority (11/12, 91.7%) of infections occurred in one province. Persistent ZIKV viremia (42 days) was found in only one woman. Six women with confirmed ZIKV infections were asymptomatic until delivery. Sequencing of 8 ZIKV isolates revealed all were of Asian lineage. All 12 ZIKV infected women gave birth to live, full-term infants; the only observed adverse birth outcome was low birth weight in one (8%) infant. Pregnancies in 3,300 ZIKV-rRT-PCR-negative women were complicated by 101 (3%) fetal deaths, of which 67 (66%) had miscarriages and 34 (34%) had stillbirths. There were no differences between adverse fetal or birth outcomes of live infants born to ZIKV-rRT-PCR-positive mothers compared to live infants born to ZIKV-rRT-PCR-negative mothers.

**Conclusions/Significance:**

Confirmed ZIKV infections occurred infrequently in this large pregnancy cohort and observed adverse maternal and birth outcomes did not differ between mothers with and without confirmed infections.

## Author summary

Zika virus (ZIKV) is an emerging infectious pathogen that was elevated to a Public Health Emergency of International Concern in 2016, due to its possible association with congenital microcephaly, a serious birth defect for infants. We launched a prospective cohort study of pregnant women to look for evidence of ZIKV infection during pregnancy. We enrolled participants in two provinces in northeastern Thailand during May 2018-January 2020. We found a low frequency of ZIKV infection (12/3312, 0.36% by rRT-PCR assay) during the study; two infections were detected at enrollment, while ten were detected later. Six of 12 ZIKV-confirmed women were asymptomatic throughout the study. Most (11/12, 92%) Zika cases occurred in one province. All 12 women with ZIKV delivered normal live births; only one infant had low birth weight and no infants had microcephaly. The eight ZIKV isolates available for whole genome sequencing were of the Asian lineage. At enrollment, the median Dengue virus IgG titer was significantly higher in ZIKV-confirmed than non-ZIKV-confirmed women. Identifying the factors associated with neonatal outcomes after ZIKV exposure *in utero* would be made easier with additional research studies.

## Introduction

Zika virus (ZIKV) is an emerging infectious pathogen that has risen to the highest level of importance in public health. ZIKV is a single-stranded ribonucleic acid (RNA) virus and is a member of the *Flavivirus* family that is primarily spread by mosquitoes [1]. ZIKV is closely related to other mosquito-borne flaviviruses including dengue virus (DENV), Japanese encephalitis virus (JEV), West Nile virus, and yellow fever virus. Patients with acute ZIKV infection often present with similar symptoms as those infected with other arboviruses, including DENV and chikungunya virus (CHIKV). Although most patients with acute ZIKV infection are asymptomatic, some patients present with mild fever, rash, arthralgia, and conjunctivitis [2].

Researchers first isolated ZIKV from the serum of a Ugandan rhesus monkey in 1947 [3]. Though serological and entomological evidence indicated that ZIKV had spread widely throughout Asia and Africa, until 2007, only a few human Zika cases were reported [4]. The two major genetic lineages for ZIKV, Asian and African, were named after the distribution of the virus in these two continents [5]. The first major ZIKV outbreaks were reported in 2007 on Yap Island in the Federated States of Micronesia and in Gabon in Central Africa [6,7]. ZIKV outbreaks in the Pacific region later occurred in close succession in French Polynesia (2013–14), New Caledonia (2014), the Cook Islands (2014), and Easter Island (2014) [8,9].

In February 2016, the World Health Organization (WHO) declared a Public Health Emergency of International Concern (PHEIC) in response to an increasing ZIKV outbreak in Latin America [10–12]. Although the link between ZIKV infection and newly reported clusters of microcephaly and other neurological disorders was unclear [13], the WHO called for increased Zika surveillance and research related to ZIKV, microcephaly, and other neurological disorders worldwide. In addition, new public health recommendations were issued to help prevent ZIKV infection in pregnant women and the general population [13,14]. Scientists have since established a causal relationship between maternal Zika virus infection during pregnancy and adverse birth outcomes in infants [14,15].

Before 2016, there had been sporadic Zika cases reported in Thailand, though researchers have recently suggested that the virus could have been circulating in the country since 2002 [16,17]. While some Zika case reports had been previously published, there were gaps in scientific understanding surrounding ZIKV infection in pregnancy in Thailand and Southeast Asia.

In response to the PHEIC declaration, the Thailand Ministry of Public Health (MOPH) and the United States Centers for Disease Control and Prevention (CDC) launched a study to investigate maternal ZIKV infection in Thailand. The Pregnancy Outcomes After Acute Zika Virus Infection in Northeastern Thailand (PrOZINT) study was a prospective cohort study designed to follow pregnant women and their newborns over time. Study objectives were to (1) estimate the incidence of ZIKV infection in pregnant women and their newborns; (2) assess the risk of adverse maternal, fetal, and infant outcomes associated with maternal ZIKV infection during pregnancy; and (3) assess the modifiers of the risk of adverse outcomes in pregnant women and infants after maternal ZIKV infection, such as gestational age at infection, previous exposure to flaviviruses, the presence of ZIKV infection symptoms, and co-infections with other flaviviruses. In this analysis, we describe the incidence of ZIKV infection and the characteristics, risk factors, and pregnancy, fetal, and infant outcomes related to ZIKV infections in a cohort of pregnant women. The results from the follow-up and developmental milestones of the infant cohort will be reported separately.

## Methods

### Ethics statement

The protocol was reviewed by the Thailand Ministry Of Public Health Ethical Committee (FWA 00001953, approval number Ref.no.22/2560) and the Institutional Review Boards of both provincial hospitals (Bueng Kan hospital, approval number BKHEC2018-06 and Mukdahan hospital, approval number MEC 05/61). Written informed consents were obtained from all participants. For participants aged 15–17 years, written informed consents were also obtained from their parent or legal guardian.

### Study setting

The PrOZINT study was conducted in the Bueng Kan and Mukdahan provinces of northeastern Thailand. These two provinces were selected due to the number of Zika cases reported via routine Zika surveillance in 2016 [18]. Both provinces are located on the border with Laos, separated by approximately 290 kilometers. Bueng Kan has a total population of 424,091 inhabitants living in eight districts. Mukdahan has a population of 353,174 living in seven districts [19]. Both provinces have a tropical climate with wet and dry seasons. Annual rainfall averages are around 2365 millimeters (mm) in Bueng Kan and 1547mm in Mukdahan [20,21].

### Study population and enrollment

From May 2018 to January 2020, pregnant women were consecutively approached for enrollment during regular antenatal visits at 134 study sites, which included the provincial hospitals and all district hospitals in Bueng Kan and Mukdahan provinces. Study staff administered an eligibility screening questionnaire and interested women signed a written informed consent form to enroll in the study. Minors (aged 15–17 years) were asked to sign an informed assent along with the consent of her parent or legal guardian. Participants were then followed from study enrollment until the end of pregnancy, with clinical touchpoints occurring during routine antenatal care (ANC) and sick visits.

### Inclusion criteria

Study staff recruited women aged 15–49 years who were visiting a participating health facility for confirmation of pregnancy or routine ANC. During the first year of the study, the eligibility criteria were confirmed pregnancy, the willingness to deliver at a participating hospital, and the intention of attending routine ANC and well-child checkups. Women with ectopic, molar, or multiple pregnancies were excluded. Women presenting to a health facility in active labor without a history of ANC were excluded from the study because it was not possible to obtain their clinical histories and records of any prior ZIKV testing. Without their previous clinical and testing information, the study would be unable to assess which outcomes of these women were ZIKV-related and the duration of viremia for any women who tested positive for ZIKV would also be unknown. Women unwilling to consent to infant or maternal laboratory testing and follow-up were not enrolled into the study.

At the beginning of the study, we enrolled pregnant women in any trimester. A review of the 2018 study data showed frequent blighted anembryonic pregnancies had occurred in several participants who were in their first trimester, resulting in several adverse events reported to the Thailand MOPH Ethical Committee. All women with this adverse pregnancy outcome had tested negative for ZIKV using rRT-PCR, and thus the causes of blighted anembryonic pregnancies for each participant were determined to be unrelated to ZIKV. In addition, the observed rate of blighted anembryonic pregnancies observed in the cohort was comparable to the background rate in this geographic area. Due to the frequent occurrences of non-ZIKV–related blighted anembryonic pregnancy, the inclusion criteria were then subsequently modified, and so only women with a gestational age ≥12 weeks were enrolled in the study. The amendment revising the inclusion criteria was approved by the Thailand Ministry Of Public Health Ethical Committee, and starting in mid-2019, only participants in their second or third trimester were enrolled in the study.

### Data collection

At enrollment and during each of the routine ANC visits, study staff administered standardized questionnaires about ZIKV risk factors, symptom history (including rash, fever, arthralgia, and conjunctivitis), and any medical history changes since the last visit. Routine ANC visits in Thailand take place at approximately weeks 12, 18, 24, 30, and 36 of gestation. Information was also abstracted from medical charts regarding routine patient history interviews and physical examinations. Variables collected included maternal demographics, risk factors and behavioral factors (such as location spent during daytime and evening and personal protective measures) for acquiring vector-borne diseases, parity, ANC clinical data, laboratory tests, *in utero* ultrasound, and clinical and laboratory data. If there was an unscheduled visit (i.e., non-ANC visit), study staff administered a questionnaire to assess for signs and symptoms related to ZIKV infection and pregnancy complications.

Maternal EDTA blood, clotted blood, and urine specimens were collected at all visits, including enrollment, routine ANC, unscheduled visits, and delivery (S1 Fig). Study staff collected 3 cubic centimeters (cc) of whole blood, 3 cc serum, and 20 cc urine from participants at enrollment, during each routine ANC visit, and each unscheduled visit to test for evidence of past or current ZIKV, DENV, and CHIKV, infection/exposure. Ultrasounds, amniocenteses, and lumbar punctures were performed if clinically indicated and according to Thailand’s guidelines [22].

ZIKV-positive women (defined as those with detectable ZIKV RNA by rRT-PCR) received follow-up prenatal assessments, clinical care, and psychosocial support according to Thailand’s guidelines for diagnosis and care of pregnant women with ZIKV infection. ZIKV-positive women were asked to return to the hospital at two-week intervals for clinical examinations and blood and urine sample collection, until two consecutive samples were negative for ZIKV by rRT-PCR. After that time, the normal frequency of routine ANC visits resumed.

The study staff administered labor and delivery case report forms to and collected whole blood, serum, and urine from mothers at delivery or within the first 96 hours after delivery. Infant EDTA blood and urine were collected at delivery as a part of the infant cohort follow-up procedures. Neonatal examination data were abstracted, including measurements of the head circumference, weight, and length, infant APGAR scores, a Dubowitz examination score (a method to estimate an infant’s gestational age by combined physical and neuromuscular assessment), and cranial ultrasound findings.

### Laboratory testing

Due to the serological cross-reactivity among flaviviruses circulating in Thailand, we then use molecular test to confirm ZIKV infection in pregnant women. In addition, to better understand background of possibility of any flavivirus infections among each enrolled pregnant women, especially if occurred between their ANC (study) visits, the serological tests were also performed at every visit.

Maternal and infant blood specimens were tested for ZIKV, CHIKV, and DENV RNA using the CDC Trioplex rRT-PCR assay [23]. Maternal urine specimens were tested for ZIKV RNA using single-plex rRT-PCR with ZIKV primers and probes as below:

Zika1087 5’-CCGCTGCCCAACACAAG-3’

Zika1108FAM 5’-AGCCTACCTTGACAAGCAGTCAGACACTCAA-3’

Zika1163c 5’-CCACTAACGTTCTTTTGCAGACAT-3’

For both blood and urine specimens, nucleic acid was extracted by MagNaPure 24 nucleic acid extraction system (Roche, USA) with MagNa Pure 24 Total NA Isolation Kit (Cat. No. 07628036001). Trioplex rRT-PCR assays were performed as previously described [23]. A positive result was defined as cycle threshold (Ct) < 38. Singleplex rRT-PCR assays were performed as previously described [24]; specimens with Ct < 38 were considered positive for ZIKV. Whole genome sequencing was conducted on ZIKV from maternal rRT-PCR positive EDTA blood specimens by next generation sequencing using the MiSeq (Illumina, USA) platform to determine the ZIKV genotype [25].

Serologic testing was done by commercial enzyme-linked immunosorbent assay (ELISA) (InBios International, USA) according to manufacturer recommendations; results were read at 450 nm using a microplate reader (Thermo Scientific Multiskan FC, USAorSpectraMax, Molecular Devices, USA). Maternal serum specimens collected at enrollment, routine ANC visits, sick visits, and at delivery were tested for IgM antibodies against ZIKV and DENV using the Zika virus IgM capture ELISA and DENV IgM Capture ELISA, respectively. (S2 Fig) We defined a positive IgM result for ZIKV and DENV as having an immune status ratio (ISR) of ≥ 1.9, and ≥ 2.84, respectively [26–28].

IgG antibodies for ZIKV and DENV were tested in all maternal sera collected at study enrollment, unscheduled visits, and delivery. IgG antibodies were also tested in maternal sera from women who were positive for ZIKV or DENV by IgM ELISA and/or Trioplex rRT-PCR, using the ZIKV IgG Capture ELISA and DENV IgG-ELISA. IgG positive ISR for ZIKV and DENV were ≥ 1.1 and ≥ 2.84, respectively [29–31]. Women with clinical signs and symptoms of CHIKV-like infection and/or who were positive for CHIKV by TrioplexrRT-PCR were tested for CHIKV IgM and IgG antibodies using CHIK IgM ELISA and CHIKIgG ELISA, with an ISR of ≥ 1.1 for both tests [32,33].

### Case definitions-

#### Acute ZIKV infection

We classified maternal Zika cases in accordance with Thai and the World Health Organization’s guidelines [22,34]. Per these guidelines, a ZIKV case was as follows:

Laboratory confirmation of recent ZIKV infection is defined as:

the presence of ZIKV RNA or antigens in serum or other samples (e.g. saliva, tissues, urine, whole blood), or
the presence of IgM antibodies against ZIKV and a PRNT90 for ZIKV with titre ≥20 and ZIKV PRNT90 titre ratio ≥ 4 compared to other flaviviruses; and exclusion of other flaviviruses [34].

For the present analysis, we defined ZIKV infections only according to the presence or absence of ZIKV RNA, as we did not perform PRNT 90 for ZIKV. Confirmed acute ZIKV infection was defined as the detection of ZIKV RNA in a laboratory specimen by rRT-PCR (blood or urine). For DENV and CHIKV, confirmed acute infection was also defined as the detection of viral RNA by Trioplex rRT-PCR in blood specimens.

#### Maternal outcomes

Maternal death was defined as the death of a woman while pregnant or within 42 days of termination of pregnancy, irrespective of the duration and site of the pregnancy, from any cause related to or aggravated by the pregnancy or its management, but not from accidental or incidental causes. Maternal complications during pregnancy e.g., preeclampsia, gestational diabetes, etc. and route of deliveries were also collected. Obstetrical complications were defined as intra- and post-partum complications or any adverse events, e.g., high blood pressure, premature rupture of membranes, placenta previa, hemorrhage, thromboembolism, etc.

#### Fetal outcomes

Intrauterine growth restriction (IUGR), or the condition in which a fetus did not achieve the expected *in utero* growth potential due to genetic or environmental factors, was defined as an estimated fetal weight below the 10th percentile. Moderate Fetal Growth Restriction (FGR) was defined as birth weight in the 3rd to 10th percentiles, and severe FGR as less than the 3rd percentile [35]. Spontaneous abortion or miscarriage was defined as a fetus at less than 20 weeks with no signs of life prior to the complete expulsion or extraction from the mother with confirmation that the fetus showed no signs of life after delivery and could not be resuscitated. Stillbirth was defined as a fetus at or after 20 weeks with no signs of life prior to the complete expulsion or extraction from the mother with confirmation that the fetus showed no signs of life after delivery and could not be resuscitated [36,37].

#### Birth outcomes

Preterm, or premature, birth was defined as birth of a liveborn infant less than 37 weeks gestation, as calculated by gestational age by a pregnancy-dating ultrasound performed at <28 weeks gestation. The birth timeframe for extreme preterm was less than 28 weeks, very preterm was 28 to less than 32 weeks, and late preterm was 32 to less than 37 weeks [38,39]. Neonatal death was defined as the death of an infant at days 0–27 of life; a death occurring between days 0–6 was defined as an early neonatal death [40]. Small for gestational age (SGA) was defined as an infant with a birth weight below the 10th percentile for gestational age [41].

Congenital microcephaly for term or mature neonates (born at 37 weeks gestation) was defined as measured head circumference (HC) within 24–72 hours of birth that is less than the 3^rd^ percentile on the Thai child growth chart (which is based on WHO Child Growth Standards) or more than 2 standard deviations (SD) below the median for gestational age and sex on Intergrowth-21 Standard for pregnant women with confirmed gestational age [42–45]. Congenital microcephaly for preterm neonates (born at less than 37 weeks of gestation) was defined as measured HC within 24–72 hours of birth that is less than the 3^rd^ percentile for gestational age and sex on the Fenton Preterm Growth Chart. Congenital microcephaly for a pregnancy loss was defined as prenatal HC that is more than 3 SD below the mean on prenatal ultrasound or postnatal HC that is less than the 3rd percentile [46]. Suspected cases of Congenital Zika Syndrome (CZS) were defined as liveborn infant, fetal death or stillbirth with congenital microcephaly or any congenital malformation of the central nervous system (e.g. hypertonia, eye abnormalities, abnormal hearing test, etc.) or congenital contractures (e.g., club foot, arthrogryposis, etc.) [47].

A composite variable was developed to account for any adverse fetal or birth outcome, defined as one or more of the following: IUGR, preterm birth, low birth weight, SGA, congenital anomaly (e.g., microcephaly, intracranial calcification documented by postnatal cranial ultrasound), or neonatal death.

### Data management and statistical analysis

Study staff collected information on hardcopy forms and/or tablets and then entered the data into a secure, password-protected Microsoft Access database for data management at the study sites (Microsoft Corporation, Redmond, Washington, USA). Quality control measures were established. Records were routinely reviewed to determine the accuracy of data entry, and discrepancies were resolved by review and re-entry of source documents. Duplicates were checked and removed and data backups were conducted frequently in accordance with data management standard operating procedures.

Sample size was calculated based on an estimated ZIKV infection prevalence of 5% for pregnant women in Thailand [18] and ±2.5% precision of the prevalence estimate. We used a Bayesian sample size calculation [48] which took into account uncertainties of the prevalence (0–10%), sensitivity (88.1–99.9%), and specificity (93–99.9%) of EUROIMMUN AG serology testing [49]. The cohort sample size needed was calculated to be 3,148 to detect the prevalence of ZIKV infection. If we were to assume a loss to follow up of 20%, then the sample size needed at enrollment is 3,478.

Categorical variables were summarized using counts and percentages, and means and standard deviations, or the medians and interquartile ranges were calculated for continuous variables. Group comparisons were carried out using chi-square or Fisher’s exact test for proportions, as appropriate, and the t-test or Wilcoxon test for continuous variables. Household income was converted to 2017 US dollar (USD) using the 2017 yearly average exchange rates (35.372 Baht to 1 USD) from the US Internal Revenue Service [50]. Exact binomial 95% confidence intervals (95% CIs) were calculated for prevalence estimates for ZIKV, DENV, and CHIKV infections. The incidence rates were calculated as the number of rRT-PCR test positive infections divided by the number of person-weeks of follow-up among those testing negative at enrollment. The estimated time of infection was defined as the midpoint between the date of the last test-negative and first test-positive visits. We requested ZIKV-positive women to return to the hospital at two-weeks intervals, until a ZIKV-positive woman had two consecutive ZIKV tests that were PCR negative. We considered the duration of ZIKV infection to be the period from the estimated time of infection until the 1st negative PCR test result. The person-weeks of follow-up were calculated from the date of enrollment to the estimated date of infection for the test positive, or to the last test date for the uninfected. We calculated 95% CIs for incidence estimates using the exact Poisson method.

Risk ratios (RR) were used as the measure of effect for the composite fetal and birth adverse outcomes. Adjusted RR (aRR) for other significant risk factors with p-values < 0.1 were estimated using log binomial regression [51]. Risk factors considered included demographics, behaviors, pregnancy history, the presence of Zika symptoms, prior infections or immunizations, and co-infections with other flaviviruses. Final variables included in the model were determined using a backwards stepdown procedure at p ≤ 0.05. Subjects with missing data were excluded in the analysis.

All statistical analyses were performed using SAS (Version 9.4; SAS Institute, Cary, North Carolina, USA) and Stata (Stata Statistical Software: Release 17. College Station, TX: StataCorp LLC).

## Results

### Overview of maternal cohort

**Fig 1:**
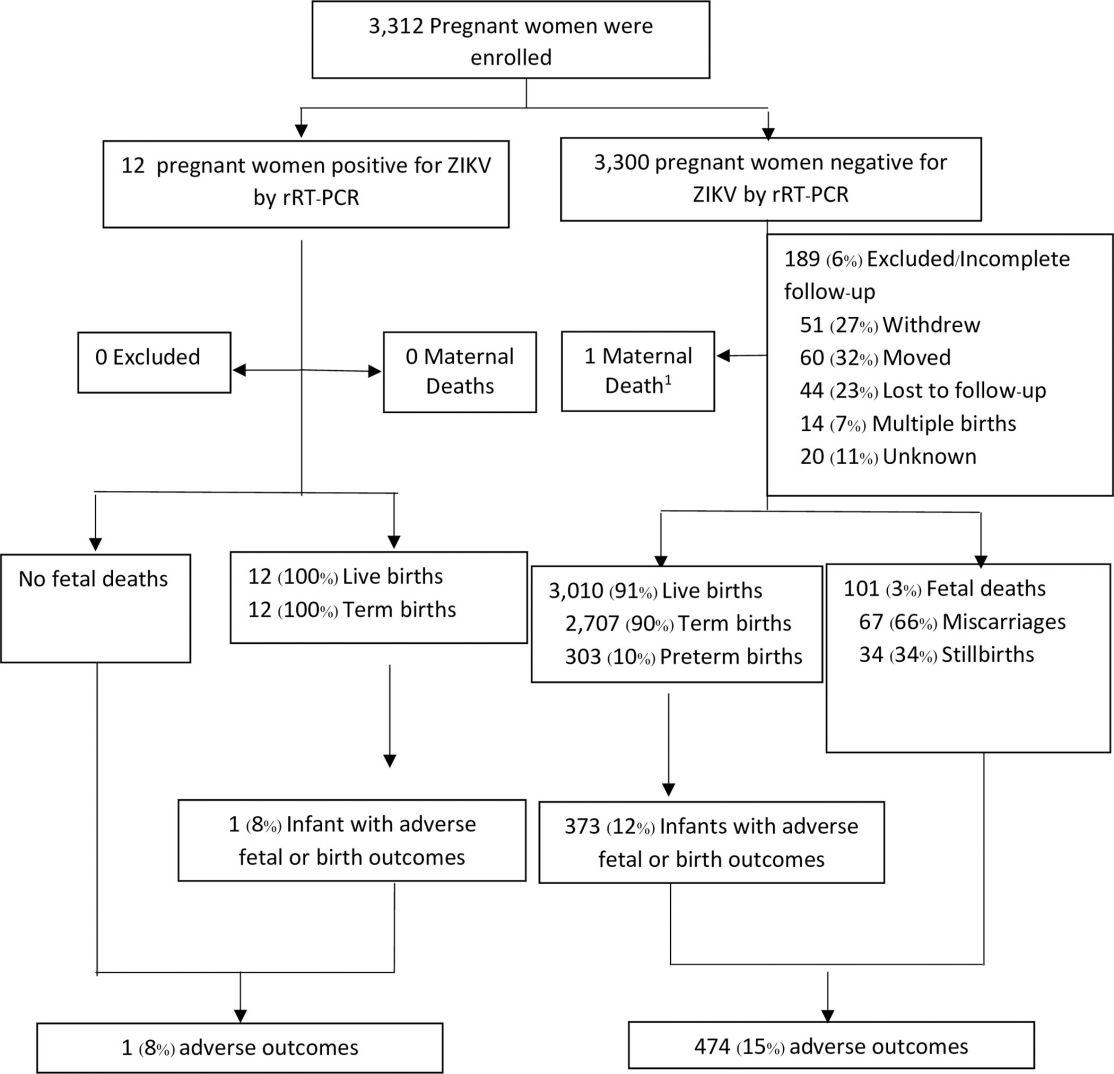
Maternal and pregnancy outcomes, Northeastern Thailand, 2018–2020. ^1^Note: One maternal participant died during childbirth. The newborn born to this deceased mother survived and is counted in the infant birth numbers.

An overview of the prospective maternal cohort and pregnancy outcomes in this study is provided in Fig 1; a total of 3,312 pregnant women were enrolled. Among the 12 ZIKV-positive women, all delivered live infants; one infant was found to have an adverse fetal outcome, specifically low birth weight (2,420 grams), resulting in a total of one (8%) adverse outcome in this group.

Incidence of ZIKV and other infections

Overall, 3,300 ZIKV-negative pregnant women were enrolled. Of these, 3,111 (94%) completed follow up, including 3,010 (91%) who had live births (2,707 term, 303 preterm) and 101 (3%) fetal losses (67 miscarriages, 34 stillbirths) (Fig 1). There were 373 infants with adverse birth outcomes born to mothers in this group, resulting in a total of 474 (15%) total adverse outcomes in this group.

From May 2018—January 2020, 12 (0.36%) of 3,312 pregnant women enrolled in the study were ZIKV rRT-PCR positive. For two women, the ZIKV rRT-PCR positive results were detected upon enrollment, while 10 (3 in 2^nd^ and 7 in 3^rd^ trimester) were detected later during the follow-up period (incidence rate of 0.15 per 1,000 person-weeks (95% CI: 0.07, 0.28)). At enrollment, 2/3312 (0.06%) participants were ZIKV-positive. The incidence rate of ZIKV infection for women enrolled based on the initial eligibility criteria including all gestational ages was ([5/48341.7]*1000 = 0.103 per 1000 weeks (95% CI: 0.034, 0.24) and ([5 /16475.4]*1000 = 0.303 per 1000 weeks (95% CI: 0.098, 0.71) (IRR = 2.93 (95% CI: 0.68, 12.75), p = 0.10) on the revised inclusion criteria of gestational age ≥12 weeks.

Ten rRT-PCR confirmed dengue infections were detected during follow-up among ZIKV rRT-PCR negative women. Dengue infection at enrollment was 1/3312 or 0.03% (95% CI: 0.0007, 0.17), with nine incident infections for an incidence rate of 0.14 per 1,000 person-weeks (95% CI: 0.06, 0.26). Chikungunya infection at enrollment was 0/3312 or 0%, with ten incident infections detected for an incidence rate of 0.15 per 1,000 person-weeks (95% CI: 0.07, 0.28). None of the confirmed Zika infections had a positive rRT-PCR result for CHIKV or DENV detected during the study.

### Distribution of ZIKV

**Fig 2:**
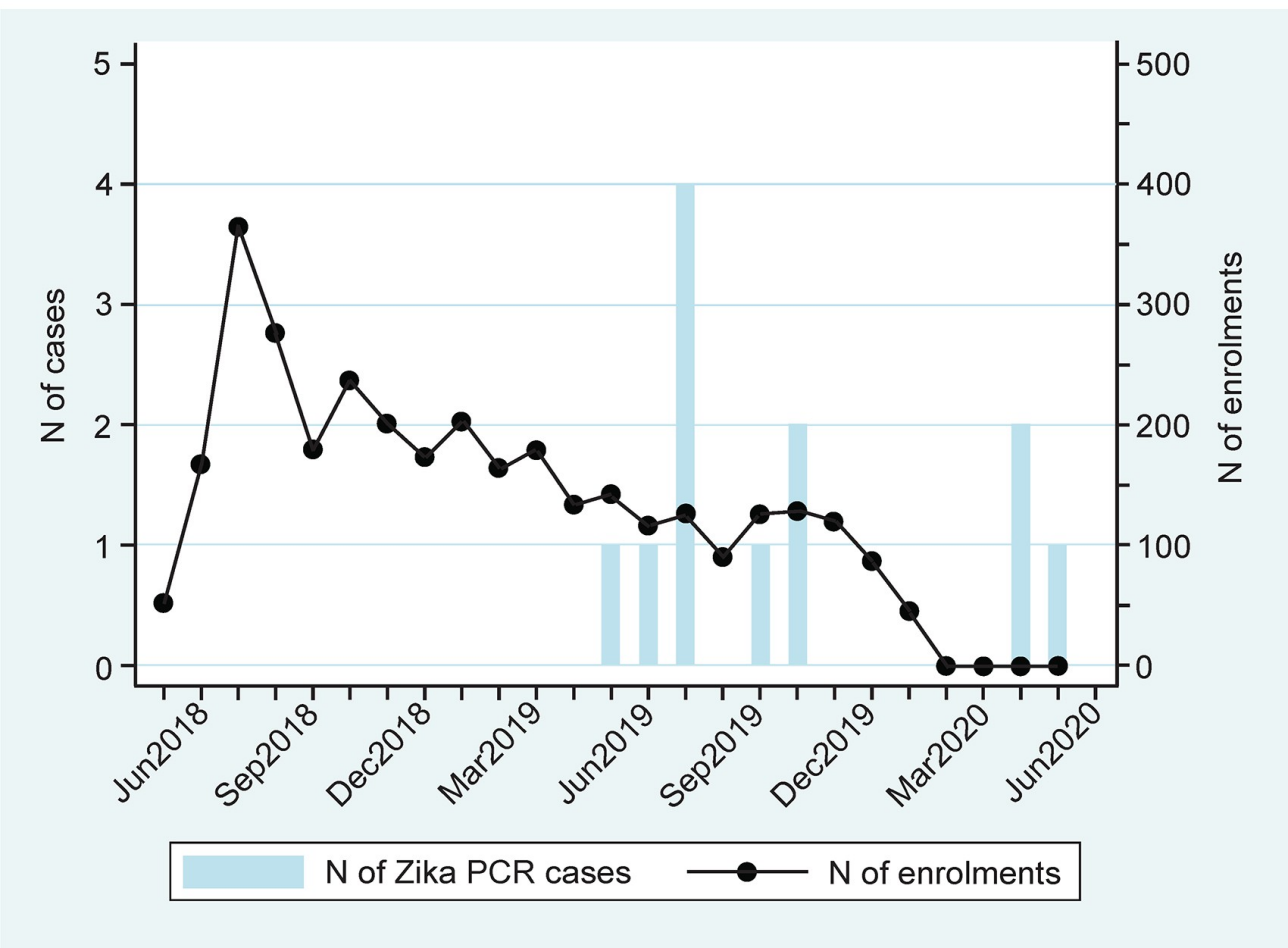
Timeline for detection of women with confirmed ZIKV infection enrolled in the study, Thailand, 2018–2020 (N = 12).

Among the 12 ZIKV-positive women, 11 were diagnosed in Bueng Kan Province and only one was detected in Mukdahan (*p* = 0.002) (Table 1). More Zika cases (n = 4) were detected in Seka District, located in the eastern part of Bueng Kan province. Nine Zika cases were detected between May to October, which is the rainy season in northeastern Thailand (Fig 2).

**Table 1:** Baseline demographic and behavioral characteristics of pregnant women at enrollment by Zika virus rRT-PCR test result (N = 3,312).

Characteristics (n, %)	Total (N = 3,312)	ZIKV rRT-PCR positive (n = 12)	ZIKV rRT-PCR Negative (n = 3,300^1^)	p-value
**Demographics**				
Age, median (Quartile 1-Quartile 3)	26 (21–32)	24.5 (19–36)	26 (21–32)	0.86
	n (%)	n (%)	n (%)	
Age groups (years)				0.31
15–20	706 (21.3)	4 (33.3)	702 (21.3)	
21–25	863 (26.1)	3 (25.0)	860 (26.1)	
26–30	794 (24.0)	1 (8.3)	793 (24.0)	
31–35	574 (17.4)	1 (8.3)	573 (17.4)	
36–40	313 (9.4)	3 (25.0)	310 (9.4)	
Greater than 40	62 (1.9)	0 (0.0)	62 (1.9)	
Province^2^				
Mukdahan	1817 (54.9)	1 (8.3)	1816 (55.0)	0.002
Bueng Kan	1495 (45.1)	11 (91.7)	1484 (45.0)	
Occupation				0.29
Agricultural	1462 (44.1%)	10 (83.4)	1452 (44.0)	
Manufacturing	36 (1.1%)	0 (0.0)	36 (1.1)	
Wholesale and resale trade	167 (5.1%)	0 (0.0)	167 (5.1)	
Public administration	121 (3.7%)	0 (0.0)	121 (3.7)	
Other	840 (25.4%)	1 (8.3)	839 (25.4)	
Unemployed	686 (20.7%)	1 (8.3)	685 (20.8)	
Education level				0.85
Elementary school or less	508 (15.3)	2 (16.7)	506 (15.3)	
Secondary	1993 (60.2)	8 (66.7)	1985 (60.2)	
Technical/ Higher	811 (24.5)	2 (16.7)	809 (24.5)	
Household income, in $US, mean (Standard Deviation)	443 (501)	402 (185)	443 (502)	0.46
**Behavioral Factors**				
Location spent during daytime^,^^4^^,^^5^				
At home	1883 (56.9)	9 (75.0)	1874 (56.8)	0.25
Away	1427 (43.1)	3 (25.0)	1424 (43.2)	
Outside	1174 (35.8)	2 (16.7)	1172 (35.9)	0.23
Inside	2102 (64.2)	10 (83.3)	2092 (64.1)	
Location spent during evening^5^^,^^6^				
At home	2120 (64.1)	9 (75.0)	2111 (64.0)	0.55
Away	1190 (35.9)	3 (25.0)	1187 (36.0)	
Outside	789 (24.1)	2 (16.7)	787 (24.1)	0.74
Inside	2489 (75.9)	10 (83.3)	2479 (75.9)	
Travel history since last antenatal care (ANC) visit ^5^^,^^7^				
Travel to another province	728 (22.0)	2 (16.7)	726 (22.0)	>0.999
No	2584 (78.0)	10 (83.3)	2574 (78.0)	
Travel to other country	13 (0.4)	0 (0.0)	13 (0.4)	>0.999
No	3299 (99.6)	12 (100.0)	3287 (99.6)	
Past vaccine history^4^				
Japanese Encephalitis (JE)	111 (3.3)	1 (8.3)	110 (3.3)	0.34
No	3201 (96.7)	11 (91.7)	3190 (96.7)	
Yellow Fever	33 (1.0)	0 (0.0)	33 (1.0)	>0.999
No	3279 (99.0)	12 (100.0)	3267 (99.0)	
Dengue	5 (0.2)	0	5 (0.2)	>0.999
No	3307 (99.8)	12 (100.0)	3295 (99.8)	
Protective measures^5^				
Use of mosquito repellents (any)	2870 (86.7)	12 (100.0)	2858 (86.6)	0.39
No	442 (13.4)	0 (0.0)	442 (13.4)	
Use of Sprays (Baygon, etc.)	630 (19.0)	4 (33.3)	626 (19.0)	0.26
No	2682 (80.1)	8 (66.7)	2674 (81.0)	
Use Coils	1378 (41.6)	8 (66.7)	1370 (41.5)	0.09
No	1934 (58.4)	4 (33.3)	1930 (58.5)	
Use topical repellant	428 (12.9)	1 (8.3)	427 (12.9)	>0.999
No	2884 (87.1)	11 (91.7)	2873 (87.1)	
Plug-in repellant	97 (2.9)	0 (0.0)	97 (2.9)	>0.999
No	3215 (97.1)	12 (100.0)	3203 (97.1)	
Bed net	2190 (66.1)	10 (83.3)	2180 (66.1)	0.36
No	1122 (33.9)	2 (16.7)	1120 (33.9)	
Long-sleeved shirt	1009 (30.5)	8 (66.7)	1001 (30.3)	0.01
No	2303 (69.5)	4 (33.3)	2299 (69.7)	
Long pants	1038 (31.3)	8 (66.7)	1030 (31.2)	0.01
No	2274 (68.7)	4 (33.3)	2270 (68.8)	
Other	49 (1.5)	0 (0.0)	49 (1.5)	>0.999
No	3263 (98.5)	12 (100.0)	3251 (98.5)	
Use of larvicides	19 (0.6)	0	19 (0.6)	>0.999
No	3293 (99.4)	12 (100.0)	3281 (99.4)	
Lifestyle^5^				
Alcohol consumption	231 (7.0)	1 (8.3)	230 (7.0)	0.58
No	3081 (93.0)	11 (91.7)	3070 (93.0)	
Drug use	12 (0.4)	0 (0.0)	12 (0.4)	>0.999
No	3300 (99.6)	12 (100.0)	3288 (99.6)	
Current smoker	17 (0.5)	0 (0.0)	17 (0.5)	>0.999
No	3295 (99.5)	12 (100.0)	3283 (99.5)	

^1^ Numbers in categories may not sum to 3,300 due to missing data

^2^ Defined as the province corresponding to the hospital from which a participant was enrolled into the study

^3^ As study participants were enrolled at participating hospitals in Mukdahan or Bueng Kan, the other variable refers to participants who resided outside of Mukdahan and Bueng Kan

^4^ The question was asked ‘in general, where are you during 6 am- 4pm?’

^5^ Totals do not add up to 100%.

^6^ The question was asked ‘in general, where are you during 4pm- 12am?’

^7^ The question was asked ‘since the last ANC visit, have you been to other provinces/another country?’

### Cohort baseline characteristics

Baseline demographic, behavioral, and clinical characteristics of the 3,312 pregnant women enrolled in the study were examined (Tables 1 and 2). Overall, the average age was 26.6 (SD 6.7) years, and most women completed secondary education (n = 1995, 60%). When comparing women with and without confirmed ZIKV, women with ZIKV infection were more likely to be from Bueng Kan (p = 0.002) (Table 1). All other demographic, behavioral, and clinical characteristics examined were similar between groups. Long-sleeved shirts and long pants significantly differed among the two groups.

**Table 2:** Baseline clinical and laboratory characteristics of pregnant women at enrollment visit (N = 3,312).

Characteristics (n, %)	Total (N = 3,312)	ZIKV rRT-PCR positive (n = 12)	ZIKV rRT-PCR Negative (n = 3,300)	p-value
**Clinical characteristics**				
	n (%)	n (%)	n (%)	
Previous pregnancy				
Yes	2253 (68.0)	6 (50.0)	2247 (68.1)	0.22
No	1059 (32.0)	6 (50.0)	1053 (31.9)	
Number of previous pregnancies				
1	1297 (57.6)	3 (50.0)	1294 (57.6)	0.49
2	670 (30.0)	3 (50.0)	667 (30.0)	
≥3	286 (12.7)	0	286 (12.7)	
Previous pregnancy outcomes^1^				
Term	1656 (74.0)	2 (33.3)	1654 (73.6)	0.046
No	597 (26.5)	4 (66.7)	593 (26.4)	
Premature births	228 (10.2)	1 (16.7)	227 (10.1)	0.47
No	2025 (89.8)	5 (83.3)	2020 (89.9)	
Abortion	495 (21.9)	2 (33.3)	493 (21.9)	0.62
No	1758 (78.0)	4 (67.7)	1754 (78.1)	
Stillbirth	27 (1.2)	0 (0.0)	27 (1.2)	>0.999
No	2226 (98.8)	6 (100.0)	2220 (98.8)	
Low birthweight	214 (9.5)	1 (16.7)	213 (9.5)	0.45
No	2039 (90.5)	5 (83.3)	2034 (90.5)	
Current medical conditions^1^				
Diabetes	11 (0.3)	0 (0.0)	11 (0.3)	>0.999
No	3301 (99.7)	12 (100.0)	3289 (99.7)	
Hypothyroidism	25 (0.8)	0 (0.0)	25 (0.8)	>0.999
No	3287 (99.3)	12 (100.0)	3275 (99.2)	
Hypertension	13 (0.4)	0 (0.0)	13 (0.4)	>0.999
No	3288 (99.6)	12 (100.0)	3287 (99.6)	
Thalassemia	70 (2.1)	0 (0.0)	70 (2.1)	>0.999
No	3242 (97.9)	12 (100.0)	3230 (97.9)	
Seizure disorder	4 (0.1)	0 (0.0)	4 (0.1)	>0.999
No	3308 (99.9)	12 (100.0)	3296 (99.9)	
Other Illness	131 (4.0)	0 (0.0)	131 (4.0)	>0.999
No	3181 (96.0)	12 (100.0)	3169 (96.0)	
Past arbovirus infection history based on self-report^1^				
Zika	4 (0.1)	0 (0.0)	4 (0.1)	>0.999
No	3308 (99.9)	12 (100.0)	3296 (99.9)	
Dengue	163 (4.9)	0 (0.0)	163 (4.9)	>0.999
No	3149 (95.1)	12 (100.0)	3137 (95.1)	
Japanese Encephalitis (JE)	4 (0.1)	0 (0.0)	4 (0.1)	>0.999
No	3308 (99.9)	12 (100.0)	3296 (99.9)	
Chikungunya	10 (0.3)	0 (0.0)	10 (0.3)	>0.999
No	3302 (99.7)	12 (100.0)	3290 (99.7)	
Presence of Zika-like symptoms at enrollment visit ^1^				
Symptomatic (≥ 1 Zika symptom)	366 (11.0)	2 (16.7)	364 (11.0)	0.63
No	2946 (89.0)	10 (83.3)	2936 (89.0)	
Fever	69 (2.1)	0 (0.0)	69 (2.1)	>0.999
No	3243 (97.9)	12 (100.0)	3231 (97.9)	
Arthralgia	112 (3.4)	1 (8.3)	111 (3.4)	0.34
No	3200 (96.6)	11 (91.7)	3189 (96.6)	
Myalgia	260 (7.8)	2 (16.7))	258 (7.8)	0.24
No	3052 (92.2)	10 (83.3)	3042 (92.2)	
Retro-orbital pain	21 (0.6)	0 (0.0)	21 (0.6)	>0.999
No	3291 (99.4)	12 (100.0)	3279 (99.4)	
Rash	43 (1.3)	0 (0.0)	43 (1.3)	>0.999
No	3269 (98.7)	12 (100.0)	3257 (98.7)	
**Laboratory Results**				
Chikungunya virus (CHIKV) PCR	10 (0.3)	0 (0.0)	10 (0.3)	>0.999
Dengue virus (DENV) rRT-PCR assay	10 (0.3)	0 (0.0)	10 (0.3)	>0.999
Positive DENV IgG at enrollment	2954 (89.2)	11 (91.7)	2943 (89.2)	>0.999
No	356 (10.8)	1 (8.3)	355 (10.8)	
Titer of DENV IgG ratio at enrollment				
Median (Quartile 1-Quartile 3)	12.50 (7.72–15.94)	16.03 (13.10–18.30)	12.46 (7.70–15.93)	0.04

Numbers within cells may not add up to total n due to missing data.

^1^Patients may have more than one category selected

### Clinical and immunological characteristics

Symptoms and flavivirus-related immunological status of the 12 ZIKV-positive women throughout their pregnancies are shown in S3 Fig. Five (42%) of the 12 confirmed ZIKV infections were detected during the second trimester and the remaining 7 were detected during the third trimester. Two confirmed ZIKV infections detected at enrollment had a gestational age of around 16–17 weeks (Cases 4 and 5). Whereas, among the 10 incident Zika cases detected after enrollment, the gestational age at the estimated time of infection was 28.5 (IQR 20–33) weeks.

Among the women with confirmed ZIKV infection, six (50%) had ZIKV detected in both blood and urine, four (33%) had ZIKV detected only in blood, and two (17%) had ZIKV detected only in urine. Persistent ZIKV viremia was observed in one Zika case (Case 8, only the first rRT-PCR is shown in S3 Fig). The first positive rRT-PCR test for Case 8 was on October 15, 2019, and her last positive rRT-PCR test was 42 days later, on November 26, 2019, with five positive rRT-PCR tests during the interval. Two women with ZIKV infection had one positive rRT-PCR result late in their pregnancy but no further ZIKV rRT-PCR testing because they delivered in non-study hospitals. ZIKV was detected in eight women who subsequently had negative rRT-PCR results at all subsequent testing time points. One Zika case had a ZIKV rRT-PCR test positive in blood but negative in urine at testing timepoint 3, followed by a negative rRT-PCR test result in blood and a positive rRT-PCR test result in urine at testing timepoint 4, followed by a negative rRT-PCR test result in both blood and urine specimens at testing timepoint 5. Among the women with confirmed ZIKV infection, nine had at least one positive ZIKV IgM ELISA result at some point during follow-up (Cases 3–8 and 10–12, S3 Fig). Cases 1, 2 and 9 had negative ZIKV IgM ELISA results. Cases 4–6 sero-reverted (from positive to negative) at least once on ZIKV IgM ELISA.

Over the course of the study (S3 Fig), six of the women with confirmed ZIKV infection (Cases 1,2,4,5,7,9) were asymptomatic at enrollment and continued to be asymptomatic for the duration of follow-up.

Four (33%) cases were symptomatic during the visit when ZIKV was detected (Cases 3, 6, 10, and 11), while three (25%) cases reported symptoms prior to the visit when ZIKV was detected (Cases 8, 11, and 12); one case (Case 11) reported symptoms both prior to and during the visit when ZIKV was detected. Symptoms reported included rash, myalgia, and arthralgia. Of the 12 confirmed Zika cases, two (17%) reported more than one Zika-like symptom at the time of the visit in which the positive specimen was collected (Cases 6 and 10). None of the women with confirmed ZIKV infection reported having a previous history of ZIKV, DENV, JEV, or CHIKV infections (Table 2). No statistical differences were seen between pregnant women with or without confirmed ZIKV infection in whether they had positive dengue IgG result at enrollment (p>0.999). However, median DENV IgG titers at baseline were higher (p = 0.042) among women with confirmed ZIKV infection when compared to women without confirmed ZIKV infection.

### Contribution of ZIKV strains

Whole genome sequencing was performed on ZIKV isolates from eight cases, and all were of Asian lineage.

### Outcomes of mothers and infants with and without ZIKV infection during pregnancy

Of the 3,312 women enrolled in the study, a total of 3,123 women completed follow-up. No differences were detected between women with and without confirmed ZIKV infection in terms of pregnancy, fetal, or birth outcomes (Table 3). No obstetric complications or adverse fetal outcomes, such as fetal loss or neonatal death, occurred in the women with confirmed ZIKV infection. One maternal death occurred in a subject who did not have ZIKV infection; this woman experienced an amniotic fluid embolism. A total of 3,022 live births occurred in the maternal cohort, 12 (0.4%) of which occurred from women with confirmed ZIKV infection. The median birthweight of the cohort was 3,030 grams, with most infants at normal birthweight. (Table 3)

**Table 3:** Outcomes of pregnancy and newborns delivered by mothers with and without confirmed Zika virus infection during pregnancy in the study (N = 3,123).

Outcomes (n, %)	Total (N = 3,123)	ZIKV rRT-PCR positive (n = 12)	ZIKV rRT-PCR Negative (n = 3,111)	p-value^1^
**Pregnancy outcomes**				
	n (%)	n (%)	n (%)	
Maternal death	1 (0.03)	0 (0.0)	1 (0.03)	>0.999
No	3040 (99.97)	12 (100.0)	3028 (99.97)	
Obstetrical complications^2^	80 (2.6)	0 (0.0)	80 (2.6)	>0.999
No	2957 (97.4)	12 (100)	2945 (97.4)	
**Fetal outcomes**				
Fetal loss	101 (3.2)	0 (0)	101 (3.2)	>0.999
No	3022 (96.8)	12 (100.0)	3010 (96.8)	
Fetal loss, trimester^3^				
First	37 (37.4%)	-	37 (37.4)	
Second	40 (40.4%)	-	40 (40.4)	
Third	22 (22.2%)	-	22 (22.2)	
Neonatal death	7 (0.2)	0 (0.0)	7 (0.2)	>0.999
No	3116(99.8)	12 (100.0)	3104 (99.8)	
IUGR (EFW < 10^th^ percentile)^4^	21 (1.0)	0 (0.0)	21 (1.0)	>0.999
No	2174 (99.0)	10 (100.0)	2164 (99.0)	
**Birth outcomes (N = 3,022)**				
Live births	**N = 3022**	**N = 12**	**N = 3010**	
Delivery				
Vaginal deliveries	1743 (57.7)	7 (58.3)	1736 (57.7)	0.34
Cesarean section	676 (22.4)	1 (8.3)	675 (22.4)	
Emergency cesarean section	600 (19.9)	4 (33.3)	596 (19.8)	
Premature				
Term (≥37 week)	2719 (90.0)	12 (100.0)	2707 (89.9)	0.62
Preterm (< 37 weeks of gestation)	303 (10.0)	0 (0.0)	303 (10.1)	
Extremely preterm (<28 weeks)	7 (2.3)	0	7 (2.3)	
Very preterm (28–32 weeks)	16 (5.3)	0	16 (5.3)	
Moderate-Late preterm (32–37 weeks)	280 (92.4)	0	280 (92.4)	
Birth weight, grams (Median, Interquartile range (IQR))	3030 (2785–3310)	3055 (2947–3757)	3030 (2783–3310)	0.28
Birth weight				
Normal	2769 (91.6)	11 (91.7)	2758 (91.6)	>0.999
Low birth weight	253 (8.4)	1 (8.3)	252 (8.4)	
Low birth weight	236 (93.3)	1 (100.0)	235 (93.2)	
Very low birth weight	14 (5.5)	0 (0.0)	14 (5.6)	
Extremely low birth weight	3 (1.2)	0 (0.0)	3 (1.2)	
Birth length, cm (Median, IQR)	52 (51–54)	52 (50.5–55)	52 (51–54)	0.59
Small for gestational age	43 (1.4)	0 (0.0)	43 (1.4)	>0.999
No	2979 (98.6)	12 (100.0)	2967 (98.6)	
Head circumference at birth, cm (Median, IQR)	33 (32–34)	32.5 (32–34.5)	33 (32–34)	0.43
Microcephaly-term (2,716 mothers)	143 (5.3)	0 (0.0)	143 (5.3)	>0.999
Microcephaly-preterm (299 mothers)	7 (2.3)	_	7 (2.3)	
Apgar score, 1 minute (Median, IQR)	9 (9, 9)	9 (9, 9.5)	9 (9, 9)	0.37
Apgar score, 5 minutes (Median, IQR)	10 (10, 10)	10 (10, 10)	10 (10, 10)	0.35
Total number with adverse fetal or birth outcomes in live births	374 (12.4)	1 (8.3%)	373 (12.4%)	>0.999
No	2648 (87.6)	11 (91.7)	2637 (87.6)	
Total number of adverse infant outcomes in live births and fetal losses	475 (15.2)	1 (8.3%)	474 (15.2)	>0.999
No	2648 (84.8)	11 (91.7)	2637 (84.8)	

^1^Test used was chi square or Fisher’s Exact test for proportions and the Wilcoxon test for continuous variables

^2^ Intra and post-partum complications, adverse events, but could be minor, high blood pressure, small for gestational age

^3^Death of an infant within 28 days of life

^4^IUGR = Intrauterine growth restriction; EFW = estimated fetal weight; IQR = Interquartile range

Among 12 infants born to mothers with confirmed ZIKV infection, neither microcephaly (S4A and S4B Fig) nor SGA was found.

In a multivariable analysis examining factors related to adverse infant outcomes, which included fetal loss and any adverse fetal or birth outcomes among live births, the only factors that were independently associated with adverse infant outcomes were age, education, travel to another province, and previous pregnancy (See Table 4 for aRR and confidence intervals). In the multivariable analysis, 1.2% (37/3123) of the data were excluded due to missing data. In our study population, maternal ZIKV infection was not associated with adverse infant outcomes.

**Table 4:** Factors associated with adverse infant outcomes in the study (N = 3,123).

Characteristics	Total (N = 3,123)	Any Adverse Infant Outcome (n = 475)	No Adverse Infant Outcome (n = 2,648)	P-value	RR (95%CI)	aRR (95%CI)
**Demographics**						
Age groups				0.016		
15–20	669	125 (18.7)	544 (81.3)		ref	
21–25	812	114 (14.0)	698 (86.0)		0.75 (0.59, 95)	
26–30	749	100 (13.3)	649 (86.7)		0.71 (0.56, 0.91)	
31–35	535	77 (14.4)	458 (85.6)		0.77 (0.60, 1.00)	
36–40	298	44 (14.8)	254 (85.2)		0.79 (0.58, 1.08)	
> 40	60	15 (25.0)	45 (75.0)		1.34 (0.84, 2.13)	
Province^1^				0.63		
Mukdahan	1719	266 (15.5)	1452 (84.5)		1.04 (0.88, 1.23)	
Buengkan	1407	209 (14.9)	1196 (85.1)		Ref	
Residence				0.42		
City	1700	264 (15.5)	1436 (84.5)		Ref	
Other districts	1394	209 (15.0)	1185 (85.0)		0.96 (0.82, 1.14)	
Other Provinces	29	2 (6.9)	27 (93.1)		0.44 (0.12, 1.70)	
Occupation				0.48		
Agricultural	1390	227 (16.3)	1163 (83.7)		Ref	
Manufacturing	36	4 (11.1)	32 (89.9)		0.68 (0.27, 1.73)	
Wholesale and resale trade	157	20 (12.7)	137 (87.3)		0.78 (0.51, 1.20)	
Public administration	106	14 (13.2)	92 (86.8)		0.81 (0.49, 1.34)	
Others	795	124 (15.6)	671 (84.4)		0.95 (0.78, 1.17)	
Unemployed	639	86 (13.4)	553 (86.5)		0.82 (0.65, 1.04)	
Education level				0.013		
Elementary school or less	483	94 (19.4)	389 (80.5)		Ref	Ref
Secondary	1883	279 (14.8)	1604 (85.2)		0.76 (0.62, 0.94)	0.77 (0.62, 0.96)
Technical/ Higher	757	102 (13.5)	655 (86.5)		0.69 (0.54, 0.90)	0.72 (0.54, 0.95)
Household income, in $US, $\bar{X}$ (SD)	3121	$434 (527)	445 (503)	0.67		
Household income ($US/baht)						
0-143/0-5000	547	108 (19.7)	439 (80.3)		Ref	Ref
143-286/5001-10000	1139	153 (13.4)	986 (86.6)		0.68 (0.54, 0.85)	0.72 (0.58, 0.91)
286-571/10001-20000	933	140 (15.0)	793 (85.0)		0.76 (0.61, 0.96)	0.86 (0.68, 1.08)
≥571/ 20,001	502	73 (14.5)	429 (85.5)		0.74 (0.56, 0.97)	0.91 (0.68, 1.21)
**Behavioral Factors**						
Location spent during daytime^2^^,^^3^						
At home (vs away)	1771	263 (14.8)	1508 (85.2)	0.55	0.95 (0.80, 1.13)	
• away	1350	211 (15.6)	1139 (84.4)		ref	
Outside (vs inside)	1117	186 (16.6)	931 (83.4)	0.10	1.16 (0.98, 1.37)	
• inside	1971	284 (14.4)	1687 (85.6)		ref	
Location spent during evening						
At home (vs away)	1984	303 (15.3)	1681 (84.7)	0.86	1.01 (0.85, 1.21)	
• away	1137	171 (15.0)	966 (85.0)		Ref	
Outside (vs inside)	755	115 (15.2)	640 (84.8)	0.98	1.00 (0.83, 1.22)	
• inside	2335	355 (15.2)	1980 (84.8)		ref	
Travel history since last ANC visit						
Travel to another province	671	80 (11.9)	591 (88.1)	0.008	0.74 (0.59, 0.93)	0.76 (0.61, 0.96)
• no	2452	395 (16.1)	2057 (83.9)		ref	ref
Travel to other country	12	2 (16.7)	10 (83.3)	0.70	1.10 (0.31, 3.90)	
• no	3111	473 (15.2)	2638 (84.8)		Ref	
Past vaccine history						
JE	107	21 (19.6)	86 (80.4)	0.22	1.30 (0.88, 1.93)	
• no	3016	454 (15.0)	2562 (85.0)		ref	
Yellow Fever	32	4 (12.5)	28 (87.5)	0.81	0.82 (0.33, 2.06)	
• no	3091	471 (15.2)	2620 (84.8)		Ref	
Dengue	5	1 (20.0)	4 (80.0)	0.56	1.32 (0.23, 7.62)	
no	3118	474 (15.2)	2644 (84.8)		Ref	
Protective measures						
Use of mosquito repellents	2719	404 (14.8)	2315 (85.1)	0.16	0.84 (0.67, 1.06)	
• no	404	71 (17.6)	333 (82.4)		Ref	
Use of larvicides	16	0 (0.0)	16 (100.0)	0.15	inf	
• no	3107	475 (15.3)	2632 (84.7)		Ref	
Lifestyle						
Alcohol consumption	220	34 (15.5)	186 (84.6)	0.92	1.02 (0.74, 1.40)	
• no	2903	441 (15.2)	2462 (84.8)		Ref	
Drug use	12	2 (16.7)	10 (83.3)	0.70	1.10 (0.31, 3.90)	
• no	3111	473 (15.2)	2638 (84.8)		Ref	
Current smoker	17	4 (23.5)	13 (76.5)	0.32	1.55 (0.66, 3.67)	
• no	3106	471 (15.1)	2635 (84.8)		ref	
**Clinical characteristics**						
Previous pregnancy	2132	292 (13.7)	1840 (86.3)	0.0007	0.74 (0.63, 0.88)	0.73 (0.59, 0.89)
• no	991	183 (18.5)	808 (81.2)		ref	ref
Parity						
1	1223	168 (13.7)	1055 (86.3)	0.86	ref	
2	641	90 (14.0)	551 (86.0)		1.02 (0.81, 1.30)	
≥3	268	34 (12.7)	234 (87.3)		0.92 (0.66, 1.31)	
Previous pregnancy outcomes						
•Term	1576	220 (13.3)	1366 (86.7)	0.40	0.90 (0.71, 1.15)	
• no	556	82 (14.7)	474 (85.3)		ref	
Premature births	214	47 (21.9)	167 (78.0)	0.002	1.71 (1.30, 2.26)	
• no	1918	245 (12.8)	1673 (87.2)		ref	
Abortion	466	67 (14.4)	399 (85.6)	0.63	1.06 (0.83, 1.37)	
• no	1666	225 (13.5)	1441 (86.5)		ref	
Stillbirth	26	4 (15.4)	22 (84.6)	0.77	1.13 (0.45, 2.79)	
• no	2106	288 (13.7)	1818 (86.3)		ref	
Low birthweight	200	47 (23.5)	153 (76.5)	<0.0001	1.85 (1.41, 2.44)	
• no	1932	245 (12.7)	1687 (87.3)		ref	
Current medical conditions						
Diabetes	11	1 (9.1)	10 (91.9)	1.0	0.60 (0.09, 3.88)	
• no	3112	474 (15.2)	2638 (84.8)		Ref	
Hypothyroidism	24	2 (8.3)	22 (91.7)	0.57	0.55 (0.14, 2.06)	
• no	3099	473 (15.3)	2626 (84.7)		Ref	
Hypertension	12	1 (8.3)	11 (91.7)	1.0	0.55 (0.08, 3.58)	
• no	3111	474 (15.2)	2637 (84.8)		Ref	
Thalassemia	66	12 (18.2)	54 (81.8)	0.49	1.20 (0.72, 2.02)	
• no	3057	463 (15.1)	2594 (84.9)		Ref	
Seizure disorder	3	0 (0.0)	3 (100.0)	1.0	inf	
• no	3120	475 (15.2)	2645 (84.8)		Ref	
Other Illness	124	24 (19.3)	100 (80.7)	0.20	1.29 (0.89, 1.86)	
• no	2999	451 (15.0)	2548 (85.0)		Ref	
Past arbovirus infection history						
Zika	4	1 (25.0)	3 (75.0)	0.48	1.65 (0.30, 9.00)	
• no	3119	474 (15.2)	2645 (84.8)		Ref	
Dengue	156	20 (12.8)	136 (87.2)	0.40	0.84 (0.55, 1.27)	
• no	2967	455 (15.3)	2512 (84.7)		Ref	
JE	4	1 (25.0)	3 (75.0)	0.48	1.65 (0.30, 9.00)	
• no	3119	474 (15.2)	2645 (84.8)		ref	
Chikungunya	10	2 (20.0)	8 (80.0)	0.65	1.32 (0.38, 4.56)	
• no	3113	473 (15.2)	2640 (84.8)		Ref	
Presence of Zika-like symptoms						
Symptomatic (≥ 1 Zika symptom)	349	62 (17.8)	287 (82.2)	0.16	1.19 (0.93, 1.52)	
• no	2774	413 (14.9)	2361 (85.1)		Ref	
Fever	66	12 (18.2)	54 (81.8)	0.49	1.20 (0.72, 2.02)	
• no	3057	463 (15.1)	2594 (84.9)		Ref	
Arthralgia	107	20 (18.7)	87 (81.3)	0.34	1.24 (0.83, 1.86)	
• no	3016	455 (15.1)	2561 (84.9)		Ref	
Myalgia	250	40 (16.0)	210 (84.0)	0.71	1.06 (0.79, 1.42)	
• no	2873	435 (15.1)	2381 (84.9)		Ref	
Retro-orbital pain	19	4 (21.1)	15 (78.9)	0.52	1.39 (0.58, 3.33)	
• no	314	471 (15.2)	2633 (84.8)		Ref	
Rash	41	8 (19.5)	33 (80.5)	0.39	1.29 (0.69, 2.41)	
• no	3082	467 (15.1)	2615 (84.9)		Ref	
Zika Infection						
Positive ZIKV RT-PCR	12	1 (8.3)	11 (91.7)	0.51	0.55 (0.08, 3.58)	
• negative	3111	474 (15.2)	2637 (84.8)		Ref	
Positive ZIKV IgM	281	42 (14.8)	239 (85.2)	0.90	0.97 (0.73, 1.31)	
negative	2842	433 (15.2)	2409 (84.8)		Ref	
Positive ZIKV IgG	334	60 (18.0)	274 (82.0)	0.14	1.21 (0.95, 1.55)	
• negative	2789	415 (14.9)	2347 (85.1)		Ref	
Positive DENV results						
DENV PCR assay	10	2 (20.0)	8 (80.0)	0.66	1.32 (0.38, 4.56)	
• negative	3113	473 (15.2)	2640 (84.8)		Ref	
DENV IgM	41	7 (17.1)	34 (82.9)	0.74	1.13 (0.57, 2.22)	
• negative	3082	468 (15.2)	2614 (84.8)		Ref	
DENV IgG	2845	433 (15.2)	2412 (84.9)	0.96	1.01 (0.75, 1.35)	
• negative	278	42 (15.1)	236 (84.9)		Ref	
Positive for any flavivirus	325	52 (15.9)	273 (84.0)	0.67	1.05 (0.81, 1.37)	
• negative	2766	418 (15.1)	2348 (84.9)		Ref	

^1^ Defined as the province corresponding to the hospital from which a participant was enrolled into the study

^2^ The question was asked ‘in general, where are you during 6 am- 4pm?’

^3^ Totals do not add up to 100%.

## Discussion

### Overall results and comparisons to results from other studies

Since the ZIKV PHEIC declaration (February to November 2016), numerous studies of pregnant women and infants around the world have reported varying rates of incidence and of symptoms related to ZIKV infections during pregnancy. From May 2018 to January 2020, this large, prospective cohort study in northeastern Thailand found that ZIKV infection was infrequently detected among pregnant women and resulted in mild symptoms and no serious adverse maternal or birth outcomes were detected. The incidence of ZIKV infection among pregnant women attending ANC visits between May 2018 to January 2020 in our cohort was <0.5% which is lower than that reported from many other prospective pregnancy cohort studies conducted previously during 2016–2017. For example, higher rates were observed among symptomatic women attending an acute febrile illness clinic in Rio de Janeiro (53%) during September 2015 to May 2016 [52], as well as women with or without symptoms included in systematic population-based monitoring in French Guiana (19%) during February to June 2016 [53], and attending clinics for high-risk pregnancies in Sao Paulo (7.7%) during March 2016 –August 2017 [54], and ANC in Peru (3.2%) during May to July 2016 [55], and Honduras (2%) during July 2016-December 2016 [56]. A study among symptomatic and asymptomatic pregnant women from Mexico during July 2016 –August 2017 revealed a similar cumulative incidence (0.31) as in our study [57].

Different factors across studies in terms of populations, inclusion criteria (e.g., existence of symptoms), timing (year) of the studies, in addition to local epidemics, likely contributed to differences in incidence rates in these cohorts. However, the low incidence in our study is consistent with relatively small numbers of ZIKV infections in Thailand at the height of the ZIKV outbreak in 2016 and 2017.[58] We also observed a higher incidence in Bueng Kan province than in the neighboring Mukdahan province, which may suggest that the Zika activity in Thailand was more localized and sporadic. There remains limited evidence as to why ZIKV incidence is relatively low in Southeast Asia and why large-scale outbreaks of ZIKV, like those observed in the Pacific and the Americas, have not been seen in Asia [16,59].

### Laboratory testing considerations

Immunological cross-reactivity between the flaviviruses, especially between DENV and ZIKV, is an issue concerning serological diagnosis [3,4]. Besides misdiagnosis as other flavivirus infections, possible explanations may include virus factors (e.g., low pathogenicity of indigenous ZIKV strain, low ZIKV viremia), host factors (e.g., ZIKV protection from previous natural or acquired immunity from other flavivirus infections or vaccinations), and environmental factors (e.g., mosquito transmissibility, no natural reservoir of ZIKV) [60–65]. More research is needed to better understand questions related to these factors.

ZIKV infection in Southeast Asia has been generally characterized by asymptomatic or mild disease, with symptoms mainly consisting of fever, muscle pain, joint pain and sometimes rash or headache; more serious symptoms have been reported less commonly [60–65]. Individuals may not seek care for mild symptoms and patients with symptoms. We found that only two (17%) ZIKV-positive pregnant women in our cohort reported more than one Zika-like symptom at the time of ZIKV detection. In 2016–2017, during the peak of the outbreak, two cases of microcephaly and/or CNS malformations cases suggestive of congenital ZIKV infections or potentially associated with ZIKV infection were reported in Thailand [58].

### Birth and neonatal outcomes in the cohort

No serious adverse birth outcomes were found in ZIKV-infected pregnant women in this study. Many studies from the Americas have reported a causal link between ZIVK infection and congenital microcephaly. In a longitudinal study in Rio de Janeiro, pregnant women with acute ZIKV infection were followed-up and 42% of 117 infants born to 116 ZIKV-positive women had abnormal clinical or brain imaging findings or both, including four infants with microcephaly [52]. A prospective cohort study of women with high-risk pregnancies in São Paulo State found that infants that were ZIKV-RT-PCR positive in the first 10 days of life had a five-fold increased risk of microcephaly overall and a ten-fold increased risk of disproportionate microcephaly [54]. However, other studies did not find evidence of vertical transmission, fetal anomalies, or congenital disease [57,60,66,67]. In our study, only one of the ZIKV-positive mothers delivered a low-birth weight infant, while all the other ZIKV-positive mothers delivered normal live births; no congenital microcephaly nor other abnormalities were reported.

We also found no significant differences in adverse neonatal outcomes among pregnant women with confirmed ZIKV infection compared with uninfected women. No significant differences in incidence or outcomes were detected before and after the change in eligibility criteria based on gestational age in our study. Other studies in Southeast Asia have also found no association between ZIKV infection during pregnancy and adverse neonatal outcomes, including microcephaly [68,69] There is still limited understanding about why the pathogenicity of ZIKV infection in Southeast Asia appears to be less severe than ZIKV infection in the Americas.

### Strain virulence and prior infection

We performed whole genome sequencing of ZIKV isolates taken from three-quarters of the Zika cases in our study and all were of the Asian lineage strain. Previous studies suggest that the African-lineage ZIKV strains cause more profound cell death in human neural progenitor (hNP) cells and neurons than Asian lineage ZIKV, while Asian lineage ZIKV isolates impair the proliferation and migration of hNP cells, and neuron maturation [70,71]. It has also been suggested that the virus circulating in Southeast Asia produces low levels of viremia [60,72] It is possible the strain of ZIKV was a contributing factor for the overall mild ZIKV outcomes of infants in our cohort who were born to mothers with confirmed ZIKV infection during pregnancy.

Whether or not a previous infection by ZIKV or another flavivirus such as DENV protects against or enhances secondary infection by a heterologous *Flavivirus* is under debate [73–76]. One study found more than 95% of pregnant Thai women had neutralizing antibodies to at least one of the four dengue virus serotypes [77]. In Thailand, where DENV is endemic and where there are active JEV vaccination campaigns with high coverage, our study revealed no statistical difference in confirmed ZIKV infection in women whether or not they had positive dengue IgG results at enrollment. However, the higher DENV IgG titers among women with than without confirmed ZIKV infection observed in our study may warrant more research to support this finding, as the interaction between previous and current flaviviruses infections is still debated. Elapsed time after previous flavivirus infection could play a role in risk of acquired infection and severity of clinical presentation of subsequent flavivirus exposure, but further study is needed. The relationship between strain virulence and prior flavivirus infection should be explored further.

### Characteristics of women with and without ZIKV infection and infant outcomes

Demographic, behavioral, and baseline clinical characteristics were mostly similar between participants with and without ZIKV infection; however, working in an agricultural occupation was also associated with increased risk of ZIKV infections. Implementing measures to reduce transmission, especially among those most at risk for infection, and educating clinicians and communities about limiting exposure during periods when ZIKV is circulating can help prevent infections. Most Zika cases in our study occurred during the rainy season and were detected in specific geographic locations. Strengthening Zika surveillance and limiting travel to ZIKV-affected areas can improve early detection and prevention of further transmission. In addition, local authorities should implement strategies to prevent or inhibit mosquito breeding to control their populations in these areas [78].

Previous studies support a possible correlation between prolonged ZIKV viremia in pregnant women and adverse neonatal outcomes [79–81]. Prolonged ZIKV viremia (42 days) was documented in only one Zika case in our study which occurred during the second trimester and the woman had a normal live birth.

Congenital ZIKV infection has been associated with serious birth defects and other complications during pregnancy. In addition, microcephaly and neurodevelopmental delays can first present years after congenital ZIKV exposure [82,83] Although we found low prevalence of ZIKV infection in our cohort, pregnant women in Thailand may still be at continued risk for ZIKV infection. A report of ZIKV seroprevalence in pregnant women attending antenatal care clinics in one hospital from May to October 2019 in Bangkok, Thailand, revealed that approximately 60% of pregnant Thai women did not have detectable levels of neutralizing antibodies against ZIKV [84]. Similarly, a post-outbreak study conducted in two communities in Southern Thailand also reported a low prevalence (12.8%) of ZIKV neutralizing antibodies in pregnant women [85]. Continued surveillance for adverse infant outcomes and neurodevelopmental delays among children would support estimating the burden of congenital ZIKV infections in Southeast Asia.

### Limitations

There were several limitations to this study. The small number of ZIKV infections detected in this study may have limited our ability to detect factors associated with ZIKV infection. The sample size calculation was based on a higher expected prevalence. The small number of ZIKV infections should be taken into account when interpreting comparisons between groups. Second, we did not enroll pregnant women during the first trimester after the first year thus we may have missed some ZIKV infections that could have occurred during this early period of gestation. Previous studies have found that maternal ZIKV infections during the first trimester can result in more adverse outcomes than infections occurring in the final two trimesters [86–89]. By not enrolling women under 12 weeks of gestation, there could have been selection bias in our study as we missed all women who potentially had a ZIKV infection early in pregnancy. Our study was limited to participants who were been attending ANC visits, and therefore we do not have data about women who did not routinely engage with the public health system during pregnancy. Our study was performed in areas where other flaviviruses like DENV were co-circulating and the cross reactivity of DENV and ZIKV infection was a challenge for interpreting the DENV and ZIKV serology [90]. We did not perform a confirmatory plaque reduction neutralization test, which is used to resolve false-positive IgM antibody results caused by non-specific reactivity [90,91]. RT-PCR was used to confirm ZIKV infection to avoid challenges in interpreting positive test results [92,93]. Also, there may have been some imperfect recall regarding symptoms occurring between ANC visits. Finally, our study took place during 2018 to 2020, after Zika cases had begun to decline globally [82] and our findings may differ from those from studies conducted at the peak of the 2016 ZIKV outbreak.

## Conclusion

This study was the first and largest prospective cohort study to investigate maternal and birth outcomes in Thailand. We found a low incidence of ZIKV and no differences in adverse maternal and birth outcomes associated with confirmed maternal ZIKV infection. Our findings are relevant for researchers, public health officials, and providers who study and provide services to pregnant women and infants in Thailand and Southeast Asia. However, more research is warranted to investigate whether the ZIKV virus strain, previous exposure to other flaviviruses, or timing of ZIKV infection during pregnancy may play a role in maternal and neonatal outcomes in Thailand.

## Supporting information

S1 Fig Molecular testing algorithm for maternal specimens. (TIF)

S2 Fig Serology testing algorithm for maternal specimens. (TIF)

S3 Fig Symptoms by flavivirus type-related immunological status in the 12 pregnant women with confirmed ZIKV infection. (TIF)

S4 Fig Head circumference measures at birth among infants born to mothers with and without confirmed ZIKV infection during pregnancy in the study. (TIF)

S1 Data Dictionary for the data sets. (XLSX)

S2 Data Data for table 1,2,3, and 4. (XLSX)

S3 Data Data for S3 Fig. (XLSX)

S4 Data Data for S4 Fig. (XLSX)
