# Supplementary figures and images for: Characteristics, risk factors, and outcomes related to Zika virus infection during pregnancy in Northeastern Thailand: A prospective pregnancy cohort study, 2018–2020
Source: PLoS Negl Trop Dis. 2024 May 17;18(5):e0012176. doi: 10.1371/journal.pntd.0012176 (PMC11139345; doi:10.1371/journal.pntd.0012176)

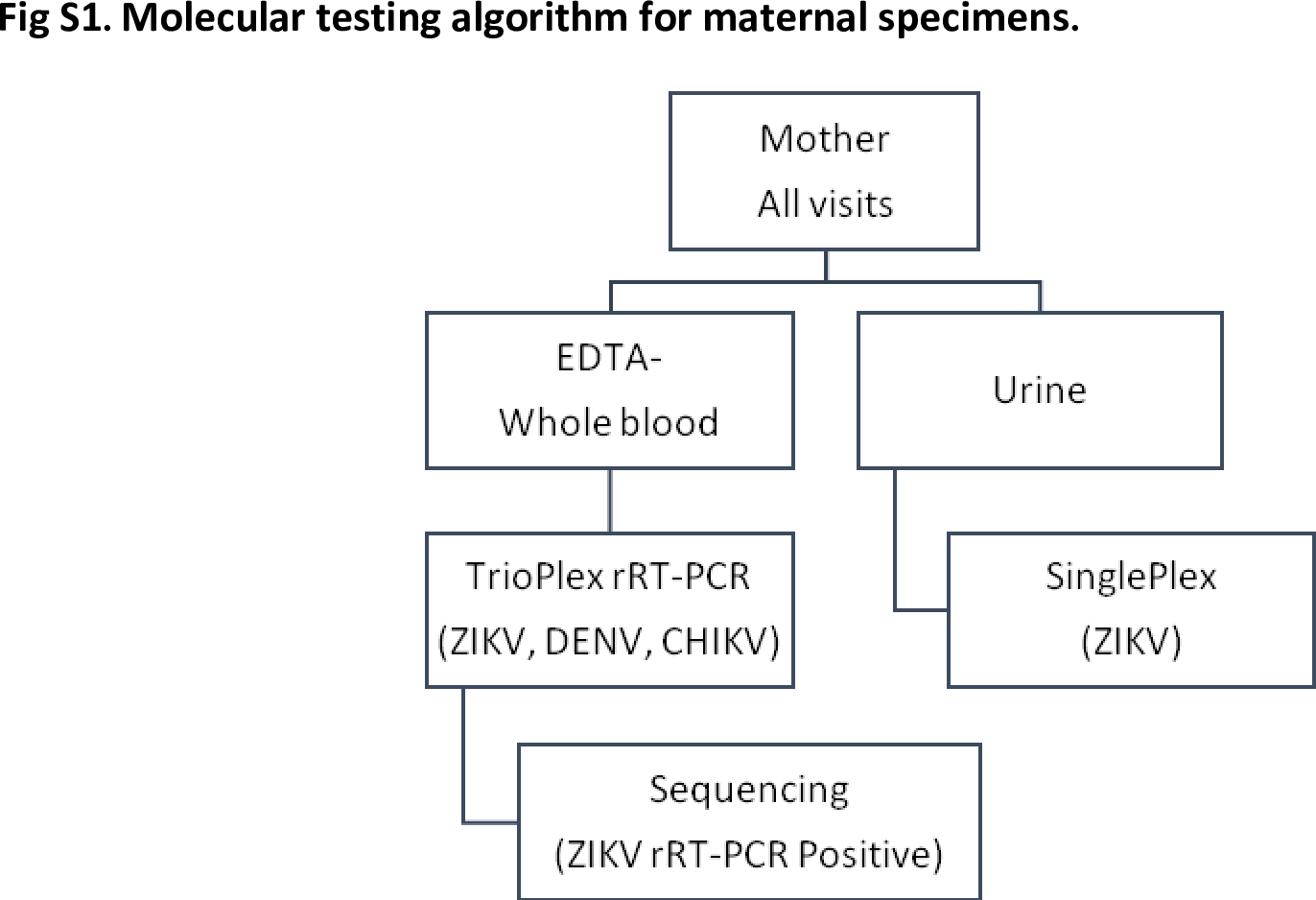

Supplement: S1 Fig — (TIF) [file pntd.0012176.s001.tif]

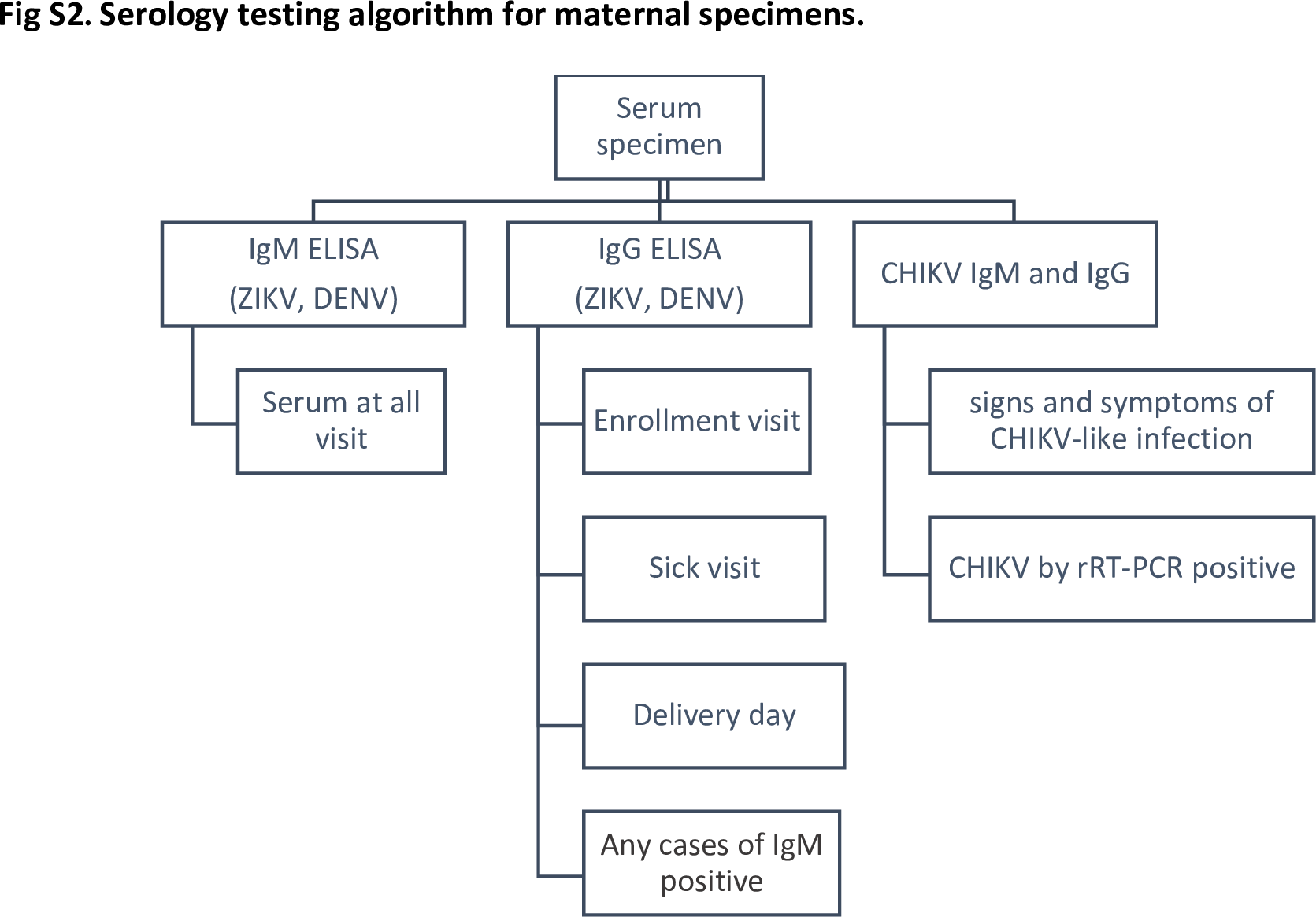

Supplement: S2 Fig — (TIF) [file pntd.0012176.s002.tif]

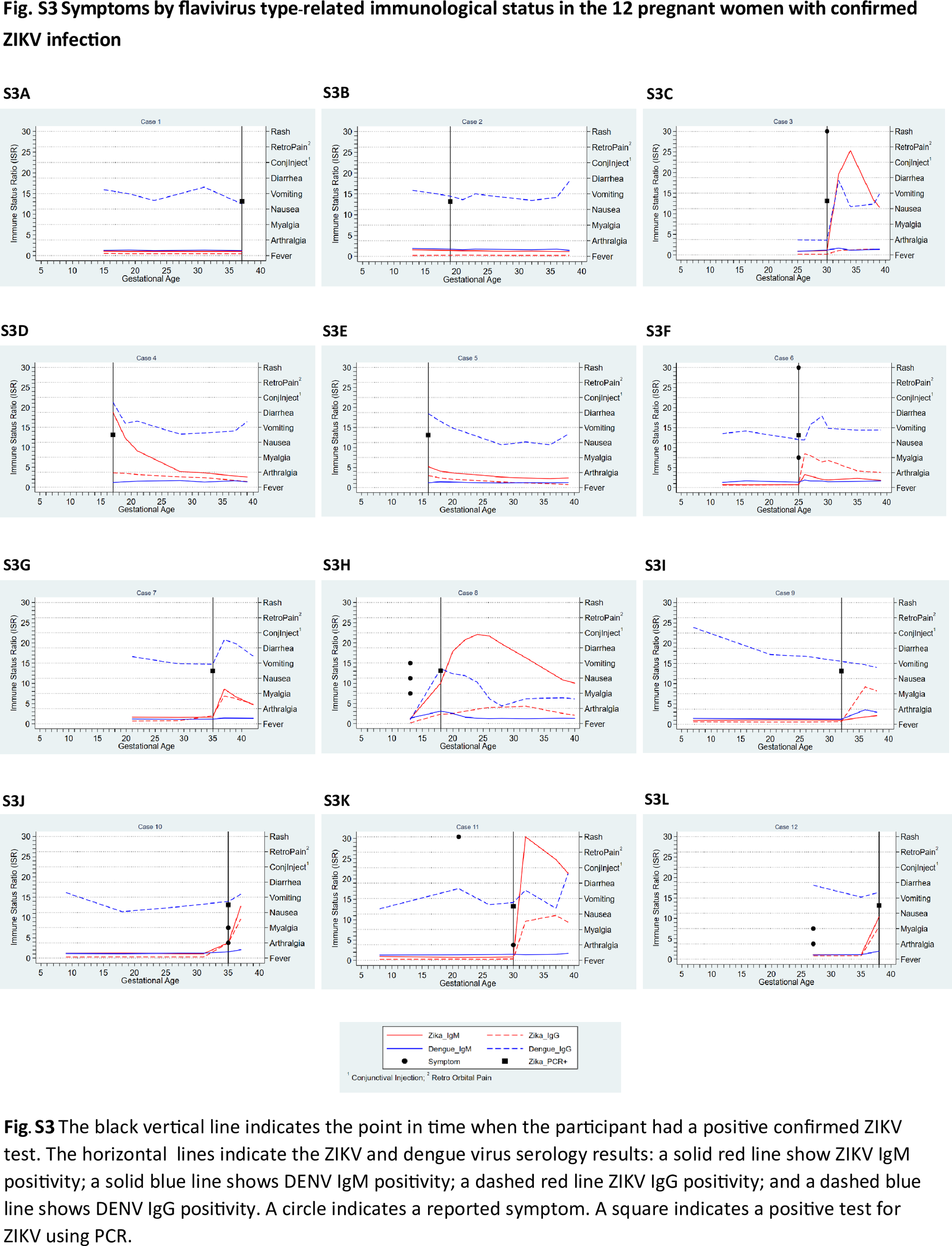

Supplement: S3 Fig — (TIF) [file pntd.0012176.s003.tif]

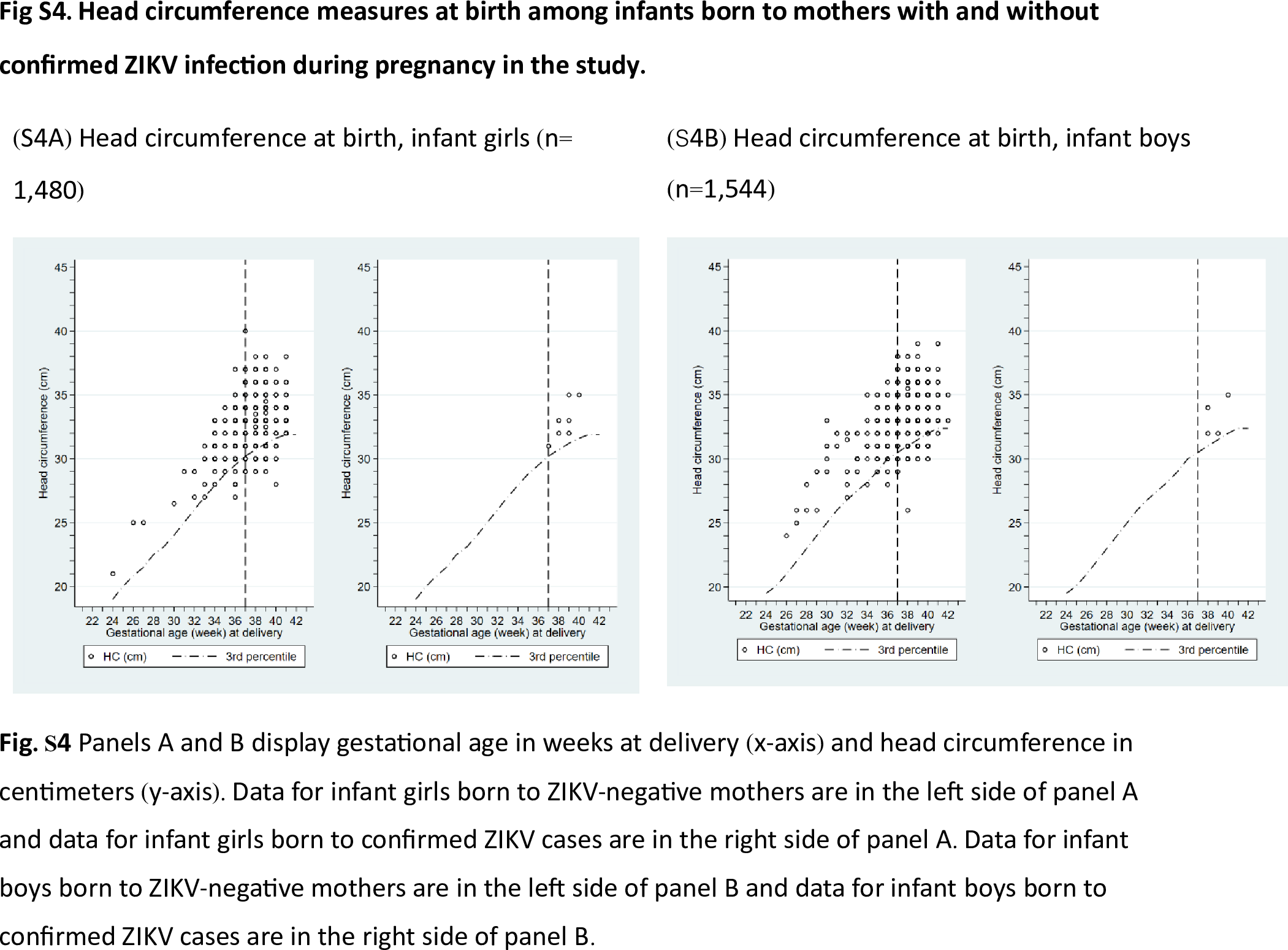

Supplement: S4 Fig — (TIF) [file pntd.0012176.s004.tif]
